# Melatonin for postoperative delirium: systematic review and meta-analysis

**DOI:** 10.1590/1806-9282.20260897

**Published:** 2026-11-09

**Authors:** Carlos Vinícius Vale de Andrade Costa, Breno Dias Lima Ribeiro, Carlos Alberto Araujo de Azevedo, Bruno Miranda Rosa Gonçalves, Ítalo Carvalho Martins, Plínio da Cunha Leal, Caio Marcio Barros de Oliveira, Ed Carlos Rey Moura

**Affiliations:** 1 Universidade Federal do Maranhão, Department of Medicine I – São Luís (MA), Brazil.

## INTRODUCTION

Postoperative delirium is a common and clinically relevant neuropsychiatric complication in older surgical patients. It is characterized by an acute and fluctuating disturbance in attention and awareness, often accompanied by changes in cognition. It is associated with higher mortality, prolonged hospital stay, cognitive decline, functional deterioration, and increased healthcare costs^1,2^. Older adults are particularly susceptible because perioperative stressors act on a background of reduced physiological reserve and pre-existing vulnerability^1,2^. Because part of the burden of delirium is potentially preventable, preventive strategies with a favorable safety profile remain important in perioperative care^3^.

The biological rationale for melatonin is credible but not, by itself, sufficient to establish clinical benefit. Delirium arises from the interaction between patient vulnerability and perioperative insults, including inflammation, oxidative stress, neurotransmitter imbalance, sleep disruption, and circadian dysregulation^2,4,5^. In the perioperative setting, anesthesia, pain, sleep disruption, and environmental changes may further disturb circadian organization^2,4,5^. Melatonin secretion declines with aging, and exogenous melatonin has circadian-regulating, anti-inflammatory, and antioxidant properties, with a generally favorable safety profile^4-6^. These features have supported its evaluation as a prophylactic intervention in older perioperative patients.

The clinical evidence, however, remains inconsistent. Previous reviews have differed in scope, with some combining surgical and non-surgical populations, melatonin and melatonin receptor agonists, and heterogeneous perioperative regimens^7-9^. Differences in population, procedure, dose, timing of administration, and delirium assessment may contribute to inconsistent estimates across studies. To address this uncertainty more narrowly, the present systematic review and meta-analysis focused on randomized controlled trials of oral melatonin in older adults undergoing surgery or invasive procedures. The objective was to evaluate the effect of oral perioperative melatonin on postoperative or early postprocedural delirium in older adults and to explore dose as a potential source of heterogeneity.

## METHODS

This systematic review and meta-analysis was conducted according to PRISMA 2020 recommendations and Cochrane methodological principles and was prospectively registered in PROSPERO (CRD420251039919)^10^.

PubMed/MEDLINE, Embase, Web of Science, Cochrane CENTRAL, and LILACS were searched from inception to March 10, 2025, without language restrictions. The search strategy combined controlled vocabulary and free-text terms related to melatonin, delirium, postoperative complications, older adults, and randomized controlled trials. Reference lists of included studies and relevant reviews were also screened.

Randomized controlled trials were eligible if they specifically enrolled older adults undergoing surgery or an invasive procedure and compared oral perioperative melatonin with placebo or standard care. For the age criterion, we accepted the definition used by each original trial when the report specified an age threshold of 60 years or older or 65 years or older, or when the study explicitly recruited geriatric or elderly patients. The primary outcome was the overall incidence of postoperative or early postprocedural delirium assessed with validated instruments or established diagnostic criteria. Studies evaluating chronic melatonin use, melatonin receptor agonists, non-oral formulations, or planned coadministration with other sedative or hypnotic agents were excluded.

Two reviewers independently screened records, assessed full texts, and extracted data on study design, population, procedure type, anesthetic approach, melatonin regimen, comparator, delirium assessment, and outcomes. Disagreements were resolved by consensus or by a third reviewer. Risk of bias was assessed independently with the Cochrane Risk of Bias 2 tool for randomized trials.

For dichotomous outcomes, RRs with 95%CIs were calculated. For continuous outcomes, mean differences (MDs) were used. Random-effects models were selected because clinical and methodological heterogeneity was expected across trials. Heterogeneity was quantified with the I^2^ statistic. Dose-based subgroup analysis was treated as exploratory because the number of studies within each subgroup was small. Publication bias was not formally assessed because fewer than 10 studies were available for the primary meta-analysis.

## RESULTS

The search identified 618 records. After removal of 229 duplicates, 389 records were screened; 375 were excluded after title and abstract review. Fourteen reports underwent full-text assessment, and six were excluded because they were ongoing, not completed, or had insufficient data. Eight randomized controlled trials met the eligibility criteria and were included in the systematic review; seven provided extractable data on overall delirium incidence and were included in the primary quantitative synthesis (Figure 1).

**Figure 1:**
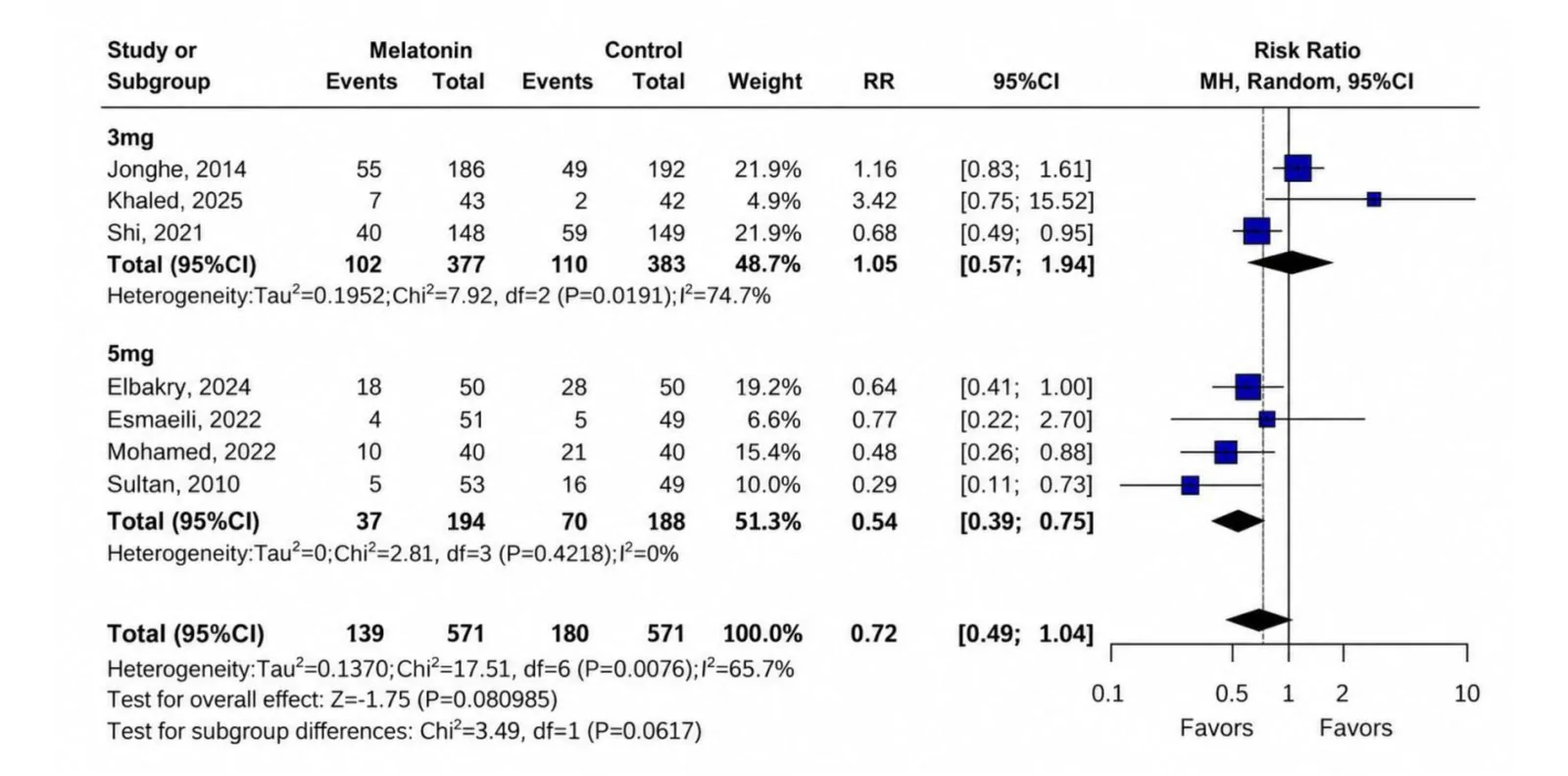
PRISMA 2020 flow diagram of study selection. Eight studies met the inclusion criteria; seven were included in the primary quantitative synthesis because one study did not report overall incidence data.

The eight trials were published between 2010 and 2025 and enrolled 1,214 participants, with 607 allocated to melatonin and 607 to control^11-18^. The procedures included hip fracture surgery or arthroplasty, orthopedic trauma surgery, colorectal surgery, mixed non-cardiac surgery, neurosurgery or general surgery, and percutaneous coronary intervention. Melatonin doses were 3 mg or 5 mg, and treatment duration ranged from preoperative administration alone to several postoperative days. Delirium was assessed with Diagnostic and Statistical Manual of Mental Disorders (DSM) criteria, Delirium Observation Screening Scale (DOSS), Confusion Assessment Method (CAM), Confusion Assessment Method for the Intensive Care Unit, 3-Minute Diagnostic Interview for CAM-defined Delirium, Nursing Delirium Screening Scale (Nu-DESC), Abbreviated Mental Test (AMT), or Mini-Mental State Examination (MMSE) (Table 1).

**Table 1:** Characteristics of included randomized controlled trials.

Study	n	Population/procedure	Anesthesia	Melatonin regimen	Delirium assessment	Overall outcome reported
Jonghe et al.^11^	378	Hip fracture surgery	Spinal/general	3 mg orally for 5 consecutive nights from admission	DSM-IV, DOSS	Melatonin 55/186 (29.6%); control 49/192 (25.5%)
Sultan^12^	102	Hip arthroplasty	Spinal	5 mg orally at bedtime and 90 min before surgery	AMT	Melatonin 5/53 (9.4%); control 16/49 (32.7%)
Shi^13^	297	Percutaneous coronary intervention	NR	3 mg/day orally for up to 7 postoperative days	CAM, CAM-ICU	Melatonin 40/148 (27.0%); control 59/149 (39.6%)
Fazel et al^14^	72	Lower limb fracture surgery; knee/hip arthroplasty	NR	5 mg orally the night before surgery, night of surgery, and two nights after surgery	NR for overall incidence	Not available for primary analysis
Mohamed et al^15^	80	Orthopedic trauma surgery	General	5 mg the night before and 30 min before surgery; then 5 mg at 21:00 for 3 postoperative days	AMT	Melatonin 10/40 (25.0%); control 21/40 (52.5%)
Esmaeili et al^16^	100	Orthopedic, neurosurgical, and general surgery	General	5 mg at bedtime before surgery and 90 min before surgery	AMT, MMSE	Day 1 AMT: melatonin 4/51 (7.8%); control 5/49 (10.2%). Day 1 MMSE: melatonin 8/51 (15.7%); control 10/49 (20.4%).
Elbakry et al^17^	100	Colorectal surgery	General	5 mg the night before surgery, 12 h before surgery, and 2 h before surgery	Nu-DESC	Melatonin 18/50 (36.0%); control 28/50 (56.0%)
Khaled et al^18^	85	Mixed non-cardiac surgery	Mostly general	3 mg 1–2 h before surgery, then bedtime to postoperative day 7 or discharge	3D-CAM, CAM-ICU	Melatonin 7/43 (16.3%); control 2/42 (4.9%)

AMT: Abbreviated Mental Test; CAM: Confusion Assessment Method; CAM-ICU: Confusion Assessment Method for the Intensive Care Unit; DOSS: Delirium Observation Screening Scale; DSM: Diagnostic and Statistical Manual of Mental Disorders; MMSE: Mini-Mental State Examination; NR: not reported; Nu-DESC: Nursing Delirium Screening Scale; 3D-CAM: 3-Minute Diagnostic Interview for CAM-defined Delirium.

Most trials were judged to be at low overall risk of bias. Three trials raised some concerns, mainly because of issues related to outcome measurement or selection of the reported result. No included trial was judged to have high risk of bias.

In the primary meta-analysis, melatonin was not associated with a statistically significant reduction in overall delirium compared with control (139/571 vs. 180/571; RR 0.72; 95%CI 0.49–1.04; p=0.081). Heterogeneity was substantial (I^2^=65.7%).

The exploratory dose subgroup analysis yielded different point estimates according to dose. In the 5 mg subgroup, the pooled estimate favored melatonin (RR 0.54; 95%CI 0.39–0.75; I^2^=0%). In the 3 mg subgroup, no clear benefit was observed (RR 1.05; 95%CI 0.57–1.94; I^2^=74.7%). The test for subgroup differences did not reach statistical significance (p=0.0617), and the finding should therefore be interpreted as hypothesis-generating rather than confirmatory (Figure 2).

**Figure 2:**
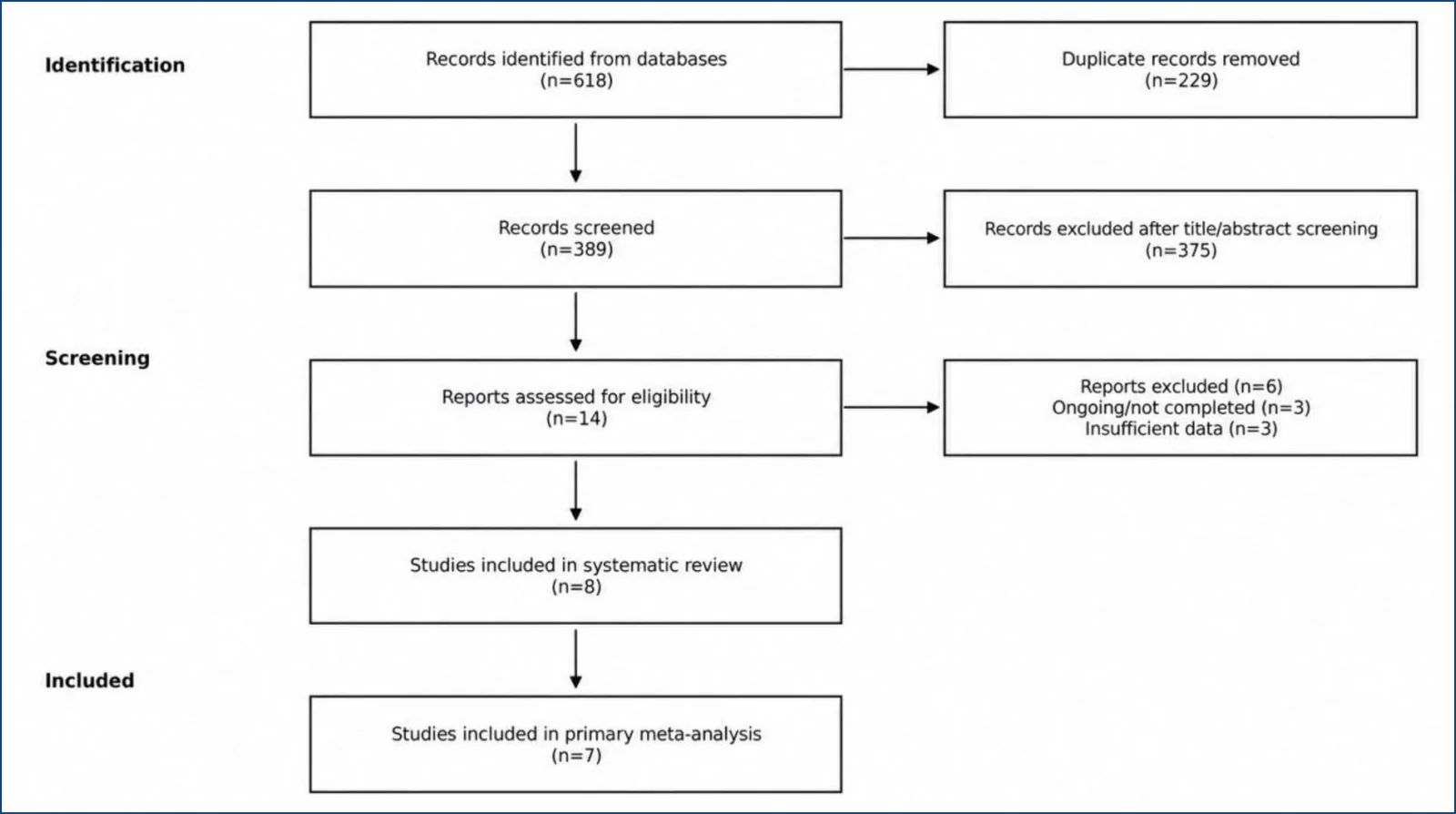
Forest plot of oral melatonin versus control for overall postoperative or postprocedural delirium, stratified by dose. Test for subgroup differences: p=0.0617; overall heterogeneity: I^2^=65.7%.

Day-specific analyses were exploratory and based on few trials. Pooled RRs were 0.99 (95%CI 0.49–2.02) in the post-anesthesia care unit, 0.47 (95%CI 0.28–0.81) on postoperative day 0, 0.49 (95%CI 0.27–0.87) on day 1, 0.44 (95%CI 0.23–0.84) on day 2, and 0.53 (95%CI 0.16–1.71) on day 3. Because these estimates came from multiple exploratory comparisons with limited study numbers, they were not interpreted as independent evidence of efficacy. Two studies assessing AMT scores showed a statistically significant but clinically small difference favoring melatonin (MD 0.18 points on a 10-point scale; 95%CI 0.04–0.32; p=0.010).

## DISCUSSION

This systematic review and meta-analysis found no statistically significant reduction in overall postoperative or early postprocedural delirium with oral melatonin in older adults. The pooled estimate was directionally favorable, but the confidence interval included no effect, and between-study heterogeneity was substantial. On this basis, the available randomized evidence does not support a definitive recommendation for routine use across all older perioperative patients.

The dose analysis requires particular caution. Although trials using 5 mg produced a more favorable pooled estimate with low within-subgroup heterogeneity, the interaction between dose subgroups was not statistically significant. The data are therefore insufficient to establish a dose–response relationship. At most, the 5 mg signal identifies a clinically plausible hypothesis that should be tested directly in trials designed for that purpose.

The heterogeneity observed in the primary analysis is clinically understandable. The included studies differed in procedure type, anesthetic approach, baseline delirium risk, timing and duration of melatonin administration, and methods of outcome ascertainment. One study enrolled patients undergoing percutaneous coronary intervention rather than conventional surgery, and another evaluated early or emergence delirium. These differences reduce the clinical uniformity of the pooled estimate and should be considered when applying the findings to surgical practice.

Outcome measurement is another important source of uncertainty. The included studies used CAM-based instruments, DSM criteria, AMT, DOSS, Nu-DESC, and MMSE. These instruments do not necessarily capture identical delirium phenotypes or severity thresholds, and the postoperative assessment windows also varied. Such variation may have contributed to the inconsistent results across postoperative days and across studies^2^.

The conclusions of this review are intentionally more conservative than those of broader syntheses that included melatonin receptor agonists, non-surgical hospitalized populations, or more heterogeneous perioperative interventions^7-9^. This narrower approach provides a more specific estimate for oral melatonin but also makes clear that the current evidence base remains small and clinically diverse.

Several limitations should be acknowledged. First, only a small number of trials were available, and seven contributed data to the primary meta-analysis. Second, the primary estimate was imprecise and accompanied by substantial heterogeneity. Third, several secondary and subgroup analyses were exploratory and should not be interpreted as confirmatory. Fourth, delirium definitions, screening instruments, and assessment windows varied across trials. Fifth, safety outcomes were not reported consistently, limiting assessment of the balance between benefit and harm. Sixth, a formal certainty-of-evidence table was not prepared. However, the presence of imprecision, inconsistency, and clinical indirectness suggests that confidence in the overall estimate would likely be limited; future updates should incorporate a structured certainty-of-evidence framework to better communicate the strength of any recommendation. Finally, the search date should be considered when interpreting the currency of the evidence base.

Future trials should use prespecified delirium definitions, standardized assessment windows, blinded outcome adjudication, and consistent safety reporting. They should also compare clinically relevant doses directly and preferentially enroll older patients at sufficiently high baseline risk, in whom a preventive intervention is more likely to show a clinically meaningful effect.

## CONCLUSION

Available randomized evidence does not establish a preventive effect of oral perioperative melatonin on postoperative or early postprocedural delirium in older adults. The current data also do not establish a difference in effect between the evaluated doses.

## Data Availability

All data analyzed in this systematic review and meta-analysis were extracted from the published studies cited in the manuscript. The study screening spreadsheet is available from the corresponding author upon reasonable request.
